# Nutz- und Bedienbarkeit einer App zur Überwindung von Sprachbarrieren im Rettungsdienst

**DOI:** 10.1007/s10049-021-00913-w

**Published:** 2021-07-02

**Authors:** Frank Müller, Eva Hummers, Jennifer Schulze, Eva Maria Noack

**Affiliations:** Institut für Allgemeinmedizin, Universitätsmedizin Göttingen, Humboldtallee 38, 37073 Göttingen, Deutschland

**Keywords:** Rettungsdienst, Migration, Geflüchteter, Benutzerfreundlichkeit, Smartphone, Emergency medical service, Language proficiency, Multilingual, Migrant, Smartphone

## Abstract

**Hintergrund:**

Im Rettungsdienst können, im Gegensatz zum stationären Setting, adäquate Dolmetscher häufig nicht ohne Weiteres hinzugezogen werden. Gleichzeitig erfordern Notfallsituationen aber eine rasche Anamnese und ein Assessment als Basis für jedes therapeutische Handeln.

**Material und Methoden:**

Eine Smartphone-App, die auf 18 Sprachen eine basale Kommunikation mittels 600 fest eingesprochener unterschiedlicher Phrasen auf 20 Sprachen ermöglicht, wurde über 6 Monate in vier Rettungswachen pilotiert. Abschließend wurde die Nutzbarkeit der App durch das gesamte Rettungsdienstpersonal in einer Fragebogenstudie unter Verwendung des System Usability Scores und des AttrakDiff-Fragebogens bewertet.

**Ergebnisse:**

Die Rücklaufquote betrug 48,5 % und *n* = 48 Fragebögen wurden ausgewertet. Das Durchschnittsalter der Befragten betrug 36 Jahre und fast zwei Drittel waren männlichen Geschlechts. Der System Usability Score zeigte im Median 67,5 Punkte, was eine grenzwertig gute Nutzbarkeit zeigte. Im AttrakDiff-Fragebogen zeigte sich die pragmatische Qualität mit durchschnittlich 0,69 (SD 0,86), die hedonische Qualität mit 0,59 (SD 0,58) und die Attraktivität (ATT) mit 0,64 Punkten (SD 0,83). Die Durchschnittswerte zeigen zufriedenstellende Werte jeweils oberhalb der neutral markierenden Grenze von 0. Auffällig zeigte sich, dass in wesentlichen Bewertungskriterien diejenigen Rettungsdienstkräfte, die angaben, die App bereits aktiv im Einsatz mit Patienten genutzt zu haben, die App signifikant besser einschätzten.

**Diskussion:**

Vor dem Hintergrund, dass es sich bei der untersuchten App um ein komplexes Arbeitswerkzeug handelt, werden die Nutzbarkeit und Attraktivität als insgesamt gut eingeschätzt, wobei in der Nutzung erfahrene Rettungskräfte diese noch positiver einschätzten. Dies könnte auf eine Art Schwellenangst hindeuten, einer bereits durch Sprach- und kulturelle Barrieren geprägten Rettungssituation mit einer ebenfalls recht komplexen Intervention zu begegnen.

## Abstract (en)

**Background:**

Communication with foreign-speaking patients in emergency medical situations can be challenging. In contrast to the inpatient setting, adequate interpreters are often not readily available in emergency services. At the same time, however, emergency situations require rapid assessment as the basis for any treatment.

**Materials and methods:**

A smartphone app that enables basic communication in 18 languages using 600 different phrases was piloted over a period of 6 months in four emergency medical service stations. Finally, the usability of the app was evaluated by the whole rescue service staff in a questionnaire study using the System Usability Score and the AttrakDiff questionnaire.

**Results:**

The response rate was 48.5% and *n* = 48 questionnaires were evaluated. The average age of the respondents was 36 years and almost two-thirds were male. The System Usability Score showed a median of 67.5 points, indicating borderline good usability. The AttrakDiff questionnaire showed pragmatic quality with an average of 0.69 (SD 0.86), hedonic quality with 0.59 (SD 0.58), and attractiveness (ATT) with 0.64 points (SD 0.83). The average values show satisfying results above the neutral limit of 0. It was observed that those rescue workers who stated that they had already actively used the app with patients rated the app significantly better.

**Discussion:**

Given that the app studied is a complex work tool, its usability and attractiveness were rated as overall good, and paramedics who had already used the app rated it even more positively. This could indicate a hesitancy by some paramedics to use a complex digital tool in complex situations that are already characterized by language and cultural barriers.

## Hintergrund

Rettungskräfte treffen zunehmend auf nicht deutschsprechende Patienten: jüngst Geflüchtete und andere Migranten, die erst wenig Deutsch können, Touristen, Saisonarbeitskräfte oder Durchreisende [1]. Bei diesen Einsätzen können Sprachbarrieren die Ersteinschätzung und Behandlung erschweren [2]. Im Projekt DICTUM Rescue wurde in einem partizipativen Prozess eine App entwickelt, die in Notfallsituation eine Basiskommunikation mit nicht deutschsprechenden Patienten ermöglicht [3–5]. Die App wurde danach auf vier Rettungswachen in Ostniedersachsen über ein halbes Jahr auf den Rettungs- und Notfalleinsatzfahrzeugen pilotiert. Nutzbarkeit, Bedienbarkeit und Attraktivität der App sind dabei entscheidende Kriterien für eine erfolgreiche Implementation und Nutzung im Rettungsdienstbereich. Dieser Artikel stellt die Ergebnisse einer Evaluation der App durch Rettungskräfte vor.

## Material und Methoden

### Die App

**Figure 1:**
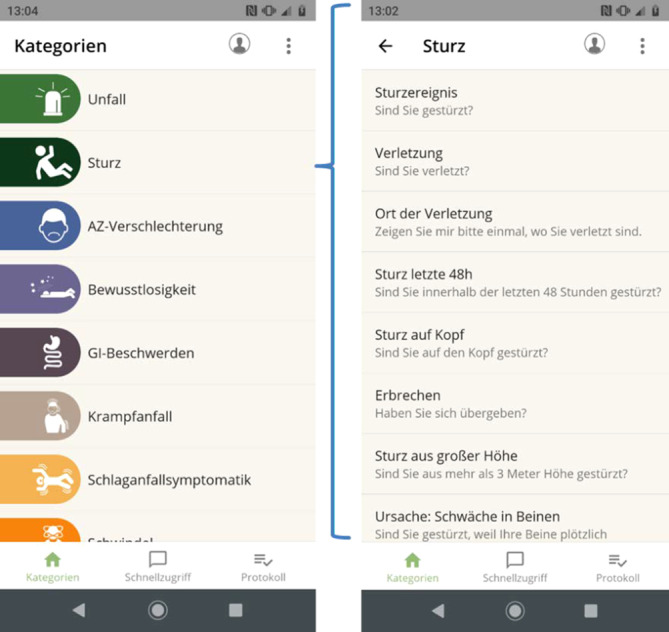

Mithilfe der entwickelten App können Rettungssanitäter fremdsprachigen Patienten situations- und symptombezogene Fragen stellen bzw. Informationen über den Rettungseinsatz, z. B. zu Untersuchungen oder getroffenen Maßnahmen, vermitteln. Die Fragen und Informationen wurden speziell für den Einsatz im Rettungsdienst entwickelt (Abb. 1). Die Inhalte sind nach Symptomen, Verdachtsdiagnosen, Organkomplexen und Rettungssituationen (z. B. Unfall, Sturz) gruppiert und innerhalb der Kategorien nach Häufigkeit und Dringlichkeit, mit der im Einsatz eine Information erhoben werden soll, geordnet. Dies soll eine schnelle Auffindbarkeit und eine am Rettungsablauf orientierte Bedienung ermöglichen. Dabei können Fragen, die bei mehreren Versorgungsszenarien relevant sind (beispielsweise die Frage nach Fieber), in mehreren Kategorien verzeichnet sein.

Zusätzlich gibt es für eine strukturierte Ersteinschätzung nach dem ABCDE-Schema („airway, breathing, circulation, disability, exposure/environment”) eine eigene Kategorie. Eine weitere Kategorie widmet sich der Abfrage von Beschwerden und Symptomen, wenn beim Eintreffen an der Rettungsstelle der subjektive Alarmierungsgrund unklar ist.

Die App unterstützt dabei 600 feste Phrasen, die in 18 Sprachen übersetzt wurden und deren Auswahl sich an den Erfordernissen im Rettungsdienst orientiert. Alle Phrasen können per Sprachausgabe – eingesprochen von professionellen Dolmetschern – wiedergegeben oder als Text angezeigt werden. Letzteres erlaubt die Verständigung mit Patienten, die schlecht hören können, oder bei sehr lauten Umgebungsgeräuschen sowie das Stellen sensibler Fragen im Beisein Dritter. Um die Antworten verstehen zu können, sind die Fragen so formuliert, dass sie mit Ja- oder Nein-Gesten oder Mimik zu beantworten sind, oder sie fordern auf, etwas zu tun oder zu zeigen, beispielsweise auf Körperregionen.

Die Inhalte der Phrasen berücksichtigen Geschlecht und Alter der Hilfesuchenden. Für Kinder als Patienten stehen kindgerechte Formulierungen zur Verfügung. Fragen über Patienten können auch an Dritte gerichtet werden, z. B. an Verwandte oder an Eltern erkrankter Kinder. Dies ist etwa hilfreich, wenn der Patient selbst nicht ansprechbar ist.

Dabei bietet die App sowohl Unterstützung zur Verständigung in alltäglichen Einsatzsituationen als auch für besondere kommunikative Situationen, z. B. im Umgang mit Angehörigen Verstorbener oder wenn Verdacht auf häusliche Gewalt besteht.

Über einen Schnellzugriff sind Fragen und Informationen zu erreichen, die in vielen Einsätzen benötigt werden, beispielsweise die Ankündigung, eine körperliche Untersuchung durchzuführen, das Einholen des Einverständnisses für einen venösen Zugang oder die Frage nach der Krankenversichertenkarte.

**Figure 2:**
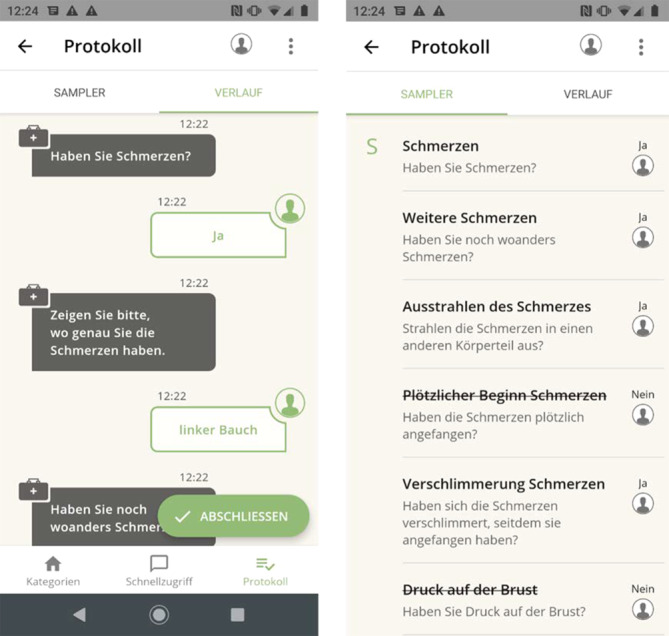

Der gesamte Gesprächsverlauf kann protokolliert werden und chronologisch in der sog. Chat-Ansicht oder sortiert nach SAMPLER-Schema („symptoms, allergies, medication, past medical history, last oral intake, events prior to incident, risk factors“) eingesehen werden (Abb. 2).

### Fragebögen

Zur Testung der Nutzbarkeit („usability“) und zur Einschätzung von Bedienung und Qualitäten der untersuchten App wurde eine Fragebogenstudie konzipiert.

Der Fragebogen umfasst drei Teile: Der erste Teil besteht aus dem AttrakDiff-Fragebogen [6], einem etablierten und validierten Fragebogen zur Testung vier unterschiedlicher Beurteilungsdimensionen: Die Dimension „pragmatische Qualität“ (PQ) zeigt dabei die Benutzbarkeit eines Produkts oder einer neuen Technologie, insbesondere wie ein Nutzer seine Ziele mithilfe dieser erreichen kann. Mit der Dimension „hedonische Qualität – Stimulation“ (HQ-S) lässt sich nachvollziehen, wie ein Produkt oder eine Technik mit neuartigen, interessanten, anregenden Funktionalitäten und Inhalten das Bedürfnis nach persönlicher Weiterentwicklung befriedigt. Die Dimension „Hedonische Qualität – Identität“ (HQ-I) misst, ob sich Nutzer mit einem neuen Produkt identifizieren. Der Fragebogenteil umfasst 28 Items, die jeweils durch zwei gegensätzliche Adjektive (z. B. „zurückweisend – einladend“) gebildet und auf einer siebenstufigen Skala angekreuzt werden. Für die vier unterschiedlichen Dimensionen wird ein jeweiliger Score errechnet, der in seinen Ausprägungen zwischen −3 (sehr negative Bewertung) und +3 (sehr positive Bewertung) liegen kann. Alle Items sind in Abb. 5 dargestellt. Der Fragebogen testet somit nicht nur die Nutzbarkeit, sondern besonders das Erleben der Bedienung, die sog. „user experience“. Pragmatische und hedonische Qualität gelten dabei in der Attraktivitätsbeurteilung als zwei voneinander unabhängige und ähnlich starke Dimensionen.

Als Zweites wurde die System Usability Scale [7] zur Nutzung der App eingesetzt, die häufig für eine grobe Evaluation der (subjektiv wahrgenommenen) Gebrauchstauglichkeit von Apps und Software herangezogen wird. Dieser validierte Fragebogen enthält 10 Fragen, die jeweils auf einer fünfstufigen Likert-Skala beantwortet werden („stimme voll zu“ – „stimme gar nicht zu“). Erfragt wird beispielsweise die Bereitschaft, die App häufig zu nutzen, oder die Notwendigkeit, Support in Anspruch nehmen zu müssen.

Im dritten Teil des Fragebogens wurden soziodemografische Basisinformationen der Befragten (Alter, Geschlecht, Berufsqualifikation) erhoben. Zudem enthielt der Teil die Fragen, ob die App bereits in Augenschein genommen wurde und ob sie bereits vom Befragten im Einsatz mit einem Patienten verwendet wurde. Abschließend gab es ein mehrzeiliges Feld, um Freitextkommentare oder Hinweise zur Verbesserung oder Problemen zu beschreiben.

### Ablauf

Die Befragung wurde in den Rettungswachen der Malteser in Braunschweig, Königslutter, Wendhausen sowie in der Rettungswache des Landkreises in Helmstedt durchgeführt. Die Fragebögen wurden zwischen dem 06.07.2020 und 08.07.2020 allen hauptamtlichen Rettungskräften und Auszubildenden der Wachen ausgeteilt bzw. in die Postfächer gelegt. Die anonymen Fragebögen sollten durch Rettungskräfte ausgefüllt und in bereitgestellte verschlossene Briefkästen bzw. versiegelte Briefumschläge gelegt werden. Die Teilnahme war freiwillig und es wurde keine Aufwandsentschädigung geboten. Die Ausgabe der Fragebögen erfolgte zu einem Zeitpunkt, zu dem die App seit 6 Monaten auf den jeweiligen Diensttelefonen der Wachen bzw. auf Smartphones in den Rettungs- und Notfalleinsatzfahrzeugen installiert und im Einsatz war. Alle Fragebögen wurden bis zum 13.10.2020 eingesammelt, der Ausfüllzeitraum wurde mit über drei Monaten so gewählt, dass alle infrage kommenden Mitarbeiter unter Berücksichtigung von Urlaubszeiten, Schichtarbeit, Krankheit oder Teilzeittätigkeit ausreichend Chance bekamen, den Fragebogen zu beantworten.

### Auswertung

Die Ergebnisse der Fragebögen wurden zu Punktscores zusammengefasst und mit korrespondierendem Median und Interquartilsabstand (IQR) angegeben. Soziodemografische Faktoren wurden mit absoluten und relativen Werten dargestellt. Einzelne Fragebogenitems des AttrakDiff-Fragebogens wurden ferner mit Mittelwert und Standardfehler des Mittelwerts bzw. in den Abbildungen mit 95 %-Konfidenzintervallen angegeben. Die interne Konsistenz der verwendeten Fragebögen wurde mittels Cronbachs α geprüft. Punktscores einzelner Items zwischen Rettungskräften, die bereits die App im Einsatz mit Patienten verwendet haben, und jenen, die dies noch nicht taten, wurden miteinander verglichen. Dafür wurde die Variable zur Frage „App bereits im Einsatz genutzt?“ als Dummy-Variable mit den Werten 0 (nicht verwendet) und 1 (verwendet) codiert und mittels Rangkorrelation nach Spearman überprüft, ob diese mit dem erzielten Punktscore korrelierten. Korrelationskoeffizienten (r) und *p*-Werte wurden angegeben, *p*-Werte ≤ 0,05 wurden als signifikant gewertet.

Alle statistischen Berechnungen wurden mit SPSS 26 durchgeführt, Abbildungen wurden mit GraphPad Prism 8.3 erstellt.

### Ethik und Datenschutz

Die Untersuchung erfolgte als Unterprojekt des DICTUM-Rescue-Projekts [1, 4]. Es besteht ein Ethikvotum der Ethikkommission der Universitätsmedizin Göttingen (9/9/18) sowie eine Übereinkunft über Datennutzung und -schutz mit den beteiligten Gebietskörperschaften sowie den Rettungsdiensten.

## Ergebnisse

### Stichprobe

**Figure 3:**
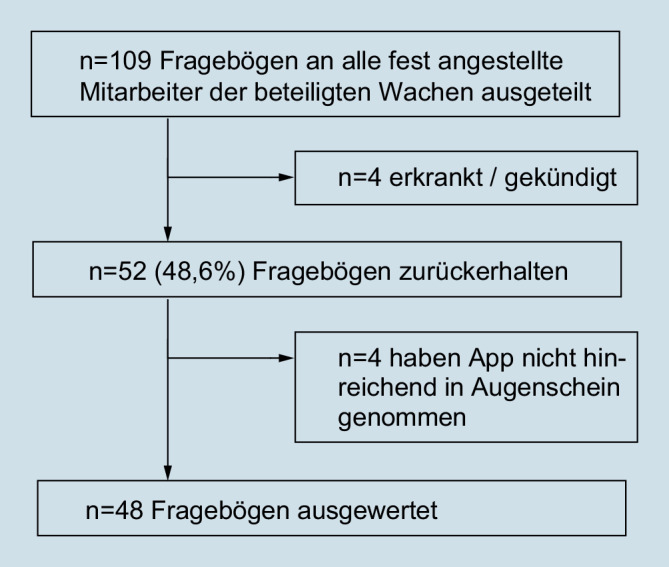

Insgesamt wurden 109 Fragebögen an alle fest angestellten Rettungsdienstmitarbeiter und Auszubildenden der im Projekt beteiligten vier Wachen ausgegeben. Vier Mitarbeiter kündigten unmittelbar nach Ausgabe der Fragebögen bzw. waren während des gesamten Rückmeldezeitraums erkrankt. 52 Fragebögen wurden zurückübermittelt, was einer Rücklaufquote von 48,6 % entspricht. Die Rücklaufquote aus den einzelnen Wachen variierte zwischen 25,6 und 83,3 %. Eine Übersicht zeigt Abb. 3.

Befragte Rettungsdienstmitarbeiter waren häufiger männlich (65,2 %), im Schnitt 36 Jahre alt. Über die Hälfte der Befragten gab an, die untersuchte App bereits selbst im Einsatz mit Patienten verwendet zu haben. Weitere Informationen zu soziodemografischen Aspekten der Befragten sind in Tab. 1 wiedergegeben.

**Table 1:** 

		*N* (%)
Geschlecht^a^	Männlich	30 (65,2)
	Weiblich	16 (34,8)
Alter (Jahre)^b^	20–29	10 (29,4)
	30–39	12 (35,3)
	40–49	9 (26,5)
	50–59	3 (8,8)
Rettungswache	Braunschweig	7 (14,6)
	Königslutter	16 (33,3)
	Wendhausen	9 (18,8)
	Helmstedt	16 (33,3)
Berufsqualifikation^c^	Rettungsassistent/-in	9 (27,3)
	Notfallsanitäter/-in	15 (45,5)
	Rettungssanitäter/-in	5 (15,2)
	Auszubildende	4 (12,1)
App bereits mit Patienten eingesetzt?	Ja	27 (56,3)
	Nein	21 (43,8)

^a^ *n* = 2 ohne Angabe; ^b^ *n* = 14 ohne Angabe, ^c^ *n* = 15 ohne Angabe

### Ergebnisse der Befragung – AttrakDiff-Fragebogen

Die pragmatische Qualität (PQ) wurde mit einem durchschnittlichen Wert von 0,69 (SD 0,86) eingeschätzt, die hedonische Qualität „Stimulation“ (HQ-S) mit 0,40 (SD 0,56), die hedonische Qualität „Identität“ (HQ-I) mit durchschnittlich 0,78 Punkten (SD 0,80) und die Attraktivität (ATT) mit 0,64 Punkten (SD 0,83). Die Durchschnittswerte zeigen insgesamt zufriedenstellende Werte jeweils oberhalb der neutral markierenden Grenze von 0. Die interne Konsistenz des Fragebogens zeigte sich als sehr gut (Cronbachs α = 0,933).

**Figure 4:**
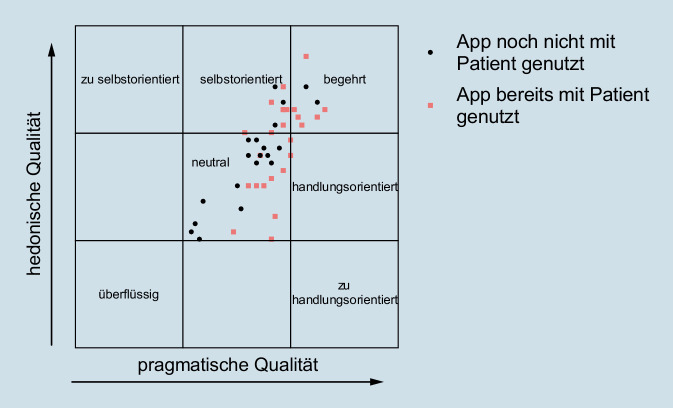

In der von den Entwicklern des AttrakDiff-Fragebogens vorgeschlagenen Portfoliodarstellung zeigt sich, dass die App zwischen neutral, begehrt und selbstorientiert rangiert (Abb. 4). Zwar fluktuieren die Einzelwerte teilweise deutlich, jedoch zeigte sich bei keinem Befragten eine Bewertung, die nahelegen würde, dass die App als überflüssig oder einseitig selbst- oder handlungsorientiert eingeschätzt wurde. Teststatistisch zeigten sich keine signifikanten Zusammenhänge zwischen Punktwerten der unterschiedlichen getesteten Dimensionen und soziodemografischen Faktoren der befragten Rettungssanitäter. Rettungskräfte, die die App bereits im Einsatz mit Patienten genutzt hatten, tendierten jedoch dazu, die App in der HQ-S-Skala deutlich besser zu bewerten (r = 0,62, *p* < 0,001).

**Figure 5:**
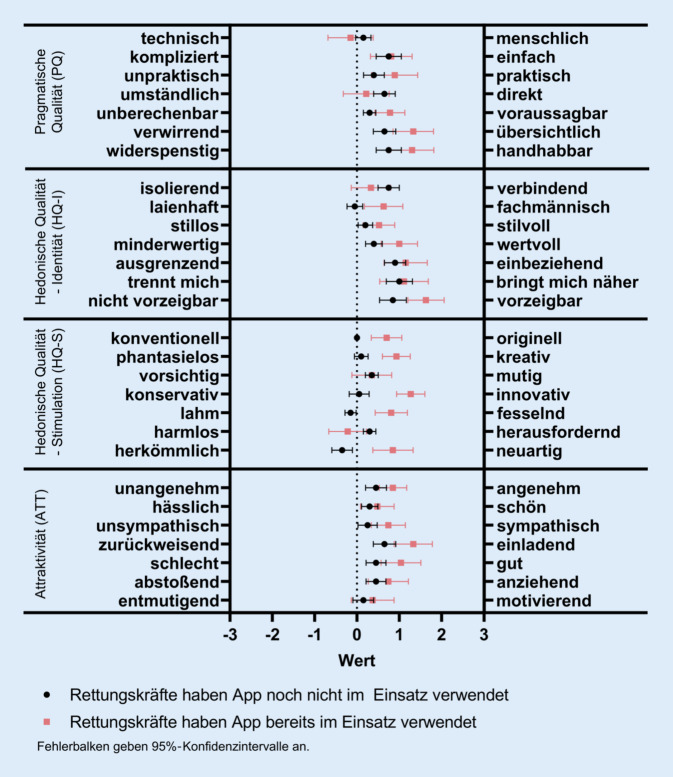

In der feineren Untersuchung auf Ebene einzelner Items konnte dieser Befund weiter differenziert werden. Befragte mit praktischer Nutzungserfahrung empfanden die App signifikant häufiger als übersichtlicher, fachmännischer, wertvoller, origineller, kreativer, innovativer, fesselnder, neuartig und besser als diejenigen, die die App noch nicht im Einsatzkontext nutzten. Eine Übersicht der einzelnen Punktscores zeigt Abb. 5.

### Ergebnisse der Befragung – System Usability Scale

Die berechnete interne Konsistenz des verwendeten Erhebungsinstruments war insgesamt gut (Cronbachs α = 0,844). Der gesamte Medianpunktwert der SUS war 67,5 Punkte (IQR 25), was nach Bangor et al. im Grenzbereich zwischen durchschnittlicher und guter Nutzbarkeit liegt [8]. Hingegen attestierten Rettungsdienstmitarbeiter, die die App bereits in der Patientenversorgung aktiv eingesetzt haben, eine durchweg gute Nutzbarkeit (Median 72,5, IQR 30). Diese Gruppen unterschieden sich insbesondere bei der Beantwortung der Fragen „Ich finde die App als unnötig komplex“ (r = −0,40, *p* = 0,005), „Ich kann mir vorstellen, dass die meisten Kollegen die App schnell zu beherrschen lernen“ (r = 0,29, *p* = 0,047), „Ich habe mich beim Einsatz der App sehr sicher gefühlt“ (r = 0,62, *p* < 0,001) und „Ich musste eine Menge Dinge lernen, bevor ich mit der App arbeiten konnte“ (r = 0,35, *p* = 0,018). In all diesen Aspekten schätzten die aktiven App-Nutzer das neue Werkzeug signifikant positiver ein als diejenigen, die die App noch nicht selbst im Einsatz am Patienten genutzt haben. Alter und Geschlecht der Befragten hatten hingegen keinen signifikanten Einfluss auf die Einschätzung. Dies traf auch auf alle einzelnen Items zu, mit Ausnahme von „Ich habe mich bei der Nutzung der App sehr sicher gefühlt“: Dieser Aussage stimmten signifikant mehr Frauen als Männer zu (r = 0,32, *p* = 0,029).

### Ergebnisse Freitextkommentare

Insgesamt haben drei Befragte die Möglichkeit zu Freitextkommentaren genutzt. Dabei wurde die Einführung einer Funktion zur Spracherkennung gewünscht, ein anderer Kommentar empfahl die Ausweitung der App-Nutzung auf Krankenhausnotaufnahmen, da hier ebenfalls ein großer Bedarf bestände. Ein dritter Kommentar berichtete darüber, dass die App einmal nicht startete. Der Fehler wurde zwischenzeitlich per Softwareupdate behoben.

## Diskussion

Die Ergebnisse zeigen, dass die untersuchte App insgesamt als gut nutzbar und attraktiv eingeschätzt wird. Insbesondere vor dem Hintergrund, dass es sich letztlich um ein „Arbeitswerkzeug“ und nicht um ein Konsumgut oder eine App mit Unterhaltungspotenzial handelt, die typischerweise mit dem verwendeten AttrakDiff-Fragebogen getestet werden, erscheint die hedonische Qualität verhältnismäßig bemerkenswert.

Bei den Ergebnissen sollten dabei die Situationen, in denen eine solche App Verwendung finden kann, stets vor Augen geführt werden: Medizinische Notfallsituationen sind häufig einschneidende und gleichsam emotional belastende Momente für Patienten und deren Angehörige. Da Rettungskräfte bei der Versorgung nochmals in besonderem Maße darauf angewiesen sind, Symptomdarstellungen, Auffindeumfeld, gesprochene Patientenaussagen, Gestik, Mimik u. v. m. zu beachten und sich ein möglichst umfassendes und stimmiges Situationsbild zu verschaffen, lässt sich annehmen, dass die Notfallversorgung und die Kommunikation über Sprach- und Kulturgrenzen hinweg als besonders herausfordernd gelten können. Die App kann dabei diese Grenzen nicht auflösen und quasi einen kommunikativen „Normalfall“ herstellen, sondern lediglich über den „Umweg“ eines digitalen Tools eine bessere Möglichkeit für das Gelingen von Kommunikation bieten. Dieser Umweg kann verständlicherweise, wie auch einzelne Befragte äußerten, eher als technisch und umständlich wahrgenommen werden, gerade weil eine solche App zunächst einmal mehr Komplexität in diese sowieso durch kommunikative Unsicherheit geprägte Situation bringt. Diese zusätzliche Komplexität lässt sich illustrieren an den 600 möglichen auswählbaren und nach Symptomgruppen und Einsatzszenarien gegliederten Phrasen, die Rettungskräften mit der App prinzipiell zur Verfügung stehen. Diese Anzahl erschien im Entwicklungsprozess als hinreichend, um eine Vielzahl von Kommunikation in Einsatzszenarien abdecken zu können, inklusive seltener, aber potenziell gefährlicher Grenzfälle. Diese hohe Anzahl sorgt auch dafür, dass es für Rettungskräfte unmöglich sein kann, über alle Frage- oder Informationsinhalte der App Bescheid zu wissen. Umso mehr ist es notwendig, dass Rettungskräfte sich ein Gefühl über den Nutzungsumfang verschaffen und sich letztlich eine Gewissheit darüber einstellt, in einem Einsatzfall eine sonst selten angewendete, aber nun dringend notwendige Phrase sicher aufzufinden. Gerade in Anbetracht dieser Funktionsfülle ist das Umfrageergebnis gerade im Hinblick auf das entwickelte User-Interface als positiv zu werten.

Auffällig ist der deutliche Unterschied in der Bewertung der App zwischen Rettungsdienstmitarbeitern, die die App bereits im Einsatz nutzten, und jenen, die dies noch nicht taten. Erstere fühlten sich sicherer in der Nutzung und empfanden sie einfacher zu bedienen. Es lässt sich folgern, dass die App im Patientenkontakt, wenn sie als Werkzeug sicher eingesetzt wurde, die Rettungssituation zu vereinfachen und abzusichern vermochte.

Gleichzeitig deutet die Bewertung auf eine Art „Schwellenangst“ derjenigen hin, die die App noch nicht nutzten. Diese könnte sich in Befürchtungen, Ängsten und abwartender Skepsis äußern. Es unterstreicht die Notwendigkeit, gerade bei digitalen Lösungen, die Funktionen bereitstellen, die in der Regel nicht alltäglich gebraucht werden, diese Schwellenängste abzubauen: Wir haben dazu etwa zu Anfang Kurzeinführungen während Teamsitzungen angeboten, bei denen Rettungskräfte in Kleingruppen spielerisch unterschiedliche Funktionen kennenlernen und die App erkunden konnten.

Limitierend muss herausgestellt werden, dass die Intervention nach einem kurzen Erprobungszeitraum von sechs Monaten evaluiert wurde und daher ein nicht unerheblicher Teil der befragten Rettungskräfte noch nicht die Möglichkeit hatte, die App im Einsatz zu testen. Wahrscheinlich konnten nur wenige Rettungskräfte die App in mehreren Einsätzen mit unterschiedlichen Erkrankungsbildern nutzen und so weitere Erfahrungen sammeln. Entsprechend zeigt die vorliegende Untersuchung ein erstes Bild, wobei weitere Untersuchungen notwendig sein werden, um auch mögliche positive patientenseitige Effekte zu bemessen.

Trotz dieser insgesamt ermutigenden Befunde zeigt sich auch Optimierungsbedarf: Eine weitere Verbesserung der Bedienbarkeit ist ein nächstes Entwicklungsziel, wobei wir durch die detaillierte Auswertung von Nutzungsdaten etwa herausfinden möchten, welche Inhalte schwer aufzufinden waren oder welche Phrasen besonders häufig genutzt wurden, und entsprechend Anpassungen in den Phrasengruppierungen vornehmen.

Gegenwärtig wird eine Funktion ergänzt, durch die die App Daten an digitale Rettungsdienstprotokollsysteme übermitteln kann. Somit besteht die Möglichkeit, Kommunikationsverläufe (inklusive Aufklärungen) mit fremdsprachigen Patienten verlässlich zu dokumentieren. Wir gehen davon aus, dass sich prognostisch die Nützlichkeit der App daher noch deutlich erhöht. Ferner ist die Lizenzierung als Medizinprodukt geplant.

Die Nichtstudienversion der App ist als „aidminutes.rescue COVID-19“ in App-Stores für Android- und iOS-Betriebssysteme verfügbar.

## Fazit für die Praxis

- Die untersuchte App wurde insgesamt als gut nutzbar und attraktiv eingeschätzt.
- Rettungskräfte, die die App bereits im Patienteneinsatz einsetzten, schätzten Nutzbarkeit und Attraktivität in wesentlichen Punkten positiver ein.
- Um Schwellenängsten bei der Verwendung von neuen digitalen Techniken zu begegnen und Bereitschaft zur Nutzung zu erhöhen, bieten sich spielerische Einführungen an.
